# Soft tissue regeneration using leukocyte-platelet rich fibrin after exeresis of hyperplastic gingival lesions: two case reports

**DOI:** 10.1186/s13256-015-0714-5

**Published:** 2015-11-02

**Authors:** A.E. di Lauro, D. Abbate, B. Dell’Angelo, G.A. Iannaccone, F. Scotto, G. Sammartino

**Affiliations:** 1 Department of Neuroscience, Reproductive and Odontostomatological Sciences, University of Naples “Federico II”, Via Pansini 5, Naples, 80131 Italy; 2 Private Practice, Oral Surgery Specialist, Naples, Italy

**Keywords:** Epulis, Hyperplastic gingival lesions, PRF, Soft tissue healing

## Abstract

**Introduction:**

Leukocyte-platelet rich fibrin belongs to a second generation of platelet concentrates that does not need biochemical blood manipulation. It is used for tissue healing and regeneration in periodontal and oral-maxillofacial surgery. We report two cases of hyperplastic gingival lesions treated by exeresis and application of leukocyte-platelet rich fibrin membranes in order to improve and accelerate tissue healing.

**Case presentation:**

Two patients (a 78-year-old Caucasian woman and a 30-year-old Caucasian man) were treated for hyperplastic gingival lesions. They underwent to exeresis of lesions and application of leukocyte-platelet rich fibrin membranes. Tissue healing was clinically evaluated after 1, 3, 7, 14 and 30 postoperative days. No recurrences were observed after 2 years of semi-annual follow up.

**Conclusions:**

We obtained rapid and good healing of soft tissues probably due to the elevated content of leukocytes, platelets and growth factors in the leukocyte-platelet rich fibrin. Based on our results we suggest the use of leukocyte-platelet rich fibrin to cover wounds after exeresis of oral neoformations such as hyperplastic gingival lesions.

## Introduction

Hyperplastic gingival lesions consist of fibro-epithelial reactions that are secondary to multiple factors: chronic inflammation, local irritation (e.g. dental plaque, tartar, root residues, projecting fillings and reconstructions, incongruous dentures), hormonal factors (puberty, pregnancy), and drugs such as anti-epileptic drugs (diphenylhydantoin sodium, valproate sodium, carbamazepine), immunosuppressive drugs (ciclosporin) and antianginal vasodilators (nifedipine) [1, 2]. A dysplastic hypothesis has been formulated regarding its onset [3, 4]; this hypothesis is suggested by the tendency of some hyperplastic gingival lesions to grow and not to regress spontaneously. They tend to recur unless a radical surgical therapy is performed. Examples of these lesions are giant-cell epulis and fibro-osseous epulis. Giant-cell epulis is the most common form and it is also called peripheral giant-cell granuloma (PGCG). It was first reported as fungus flesh in 1848 [5], then reported as giant cell reparative granuloma in 1953 [6]. This lesion is the peripheral opponent form of central giant-cell intraosseous tumors, characterized by the presence of giant cells that seem to originate from osteoclasts in the periodontal ligament [7]. Fibro-osseous epulis or peripheral ossifying fibroma (POsF) was first reported and described as ‘alveolar exostosis’ in 1844 [8]. It presents a dense collagenous stroma in which thin trabecular bone can be observed.

We report two cases of hyperplastic gingival lesions treated by exeresis and application of leukocyte-platelet rich fibrin (L-PRF) membranes in order to improve and accelerate tissue healing. L-PRF belongs to a second generation of platelet concentrates which does not need biochemical blood manipulation [9, 10]. It is used for tissue healing [11, 12] and regeneration in periodontal and oral-maxillofacial surgery [13] because of its elevated content of leukocytes, platelets and growth factors.

## Case presentation

### Case number one

**Fig. 1:**
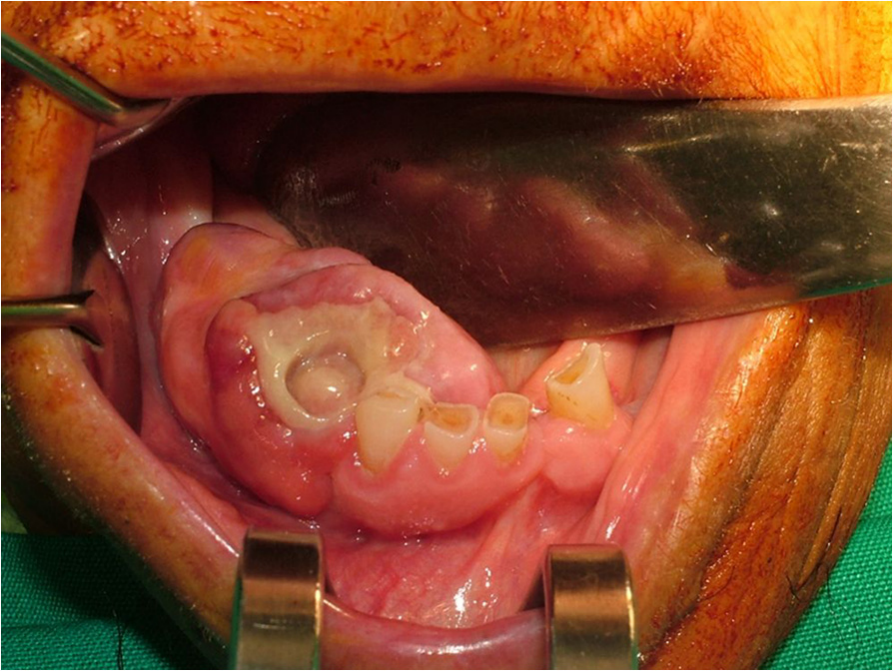
Intraoral appearance of the lesion, Patient 1

**Fig. 2:**
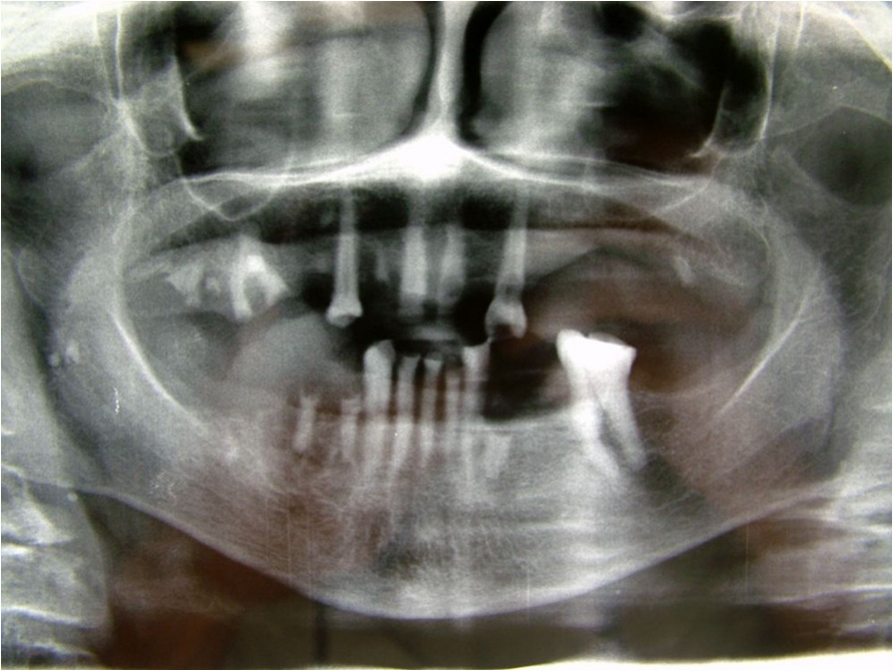
Orthopantomography, Patient 1

A 78-year-old Caucasian woman presented with a complaint of a swelling in her right mandibular molar teeth area that was painful at mastication. The swelling had arisen 4 months previously and had grown quickly. On extraoral examination she presented a right facial swelling and the skin of her face was healthy. Her regional lymph nodes were not palpable. On intraoral examination she showed poor oral hygiene. A 45×30×20mm sessile, lobular, soft tissue mass was evident in her right posterior mandibular gingiva (Fig. 1). The mucosal covering of the lesion looked rosy-purple with a focal area of ulceration. The lesion was painful and bleeding on palpation with a parenchymatous consistency. At orthopantomography two premolar residual roots were observed in the area of the lesion (Fig. 2) with no other significant findings.

### Case number two

**Fig. 3:**
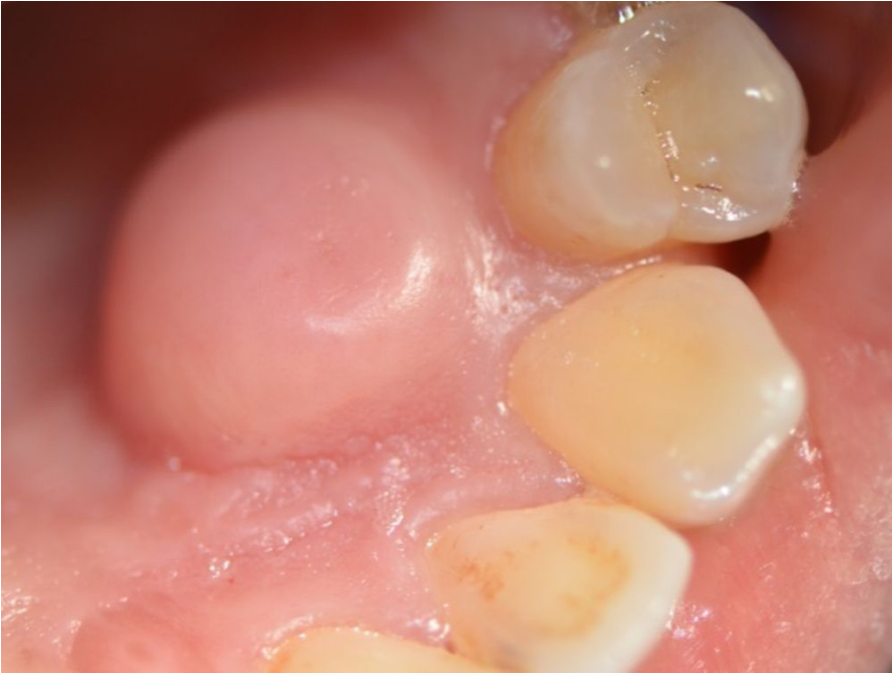
Intraoral appearance of the lesion, Patient 2

A 30-year-old Caucasian man presented with a complaint of a swelling in his left hard palate region which had arisen approximately 2 months previously after contact with hot food. The lesion was not painful but was responsible for discomfort on swallowing and phonation. On extraoral examination facial skin lesions or swellings were not highlighted and his regional lymph nodes were not palpable. An intraoral examination showed a sessile and detected lesion of hard palate in correspondence with left maxillary lateral incisor, left maxillary canine and left maxillary first premolar. The lesion was characterized by a diameter of 10mm and the mucosa which covered it looked eutrophic (Fig. 3). The lesion was not painful on palpation and it had a hard consistency. He showed good level of oral hygiene. The adjacent teeth did not have carious or periodontal lesions and they responded positively to the vitality test. At orthopantomography no signs of pathology were found.

### Surgical procedures

In both cases we decided to proceed with a surgical approach characterized by complete excision of lesions and application of L-PRF membranes in order to improve and accelerate tissue healing.

**Fig. 4:**
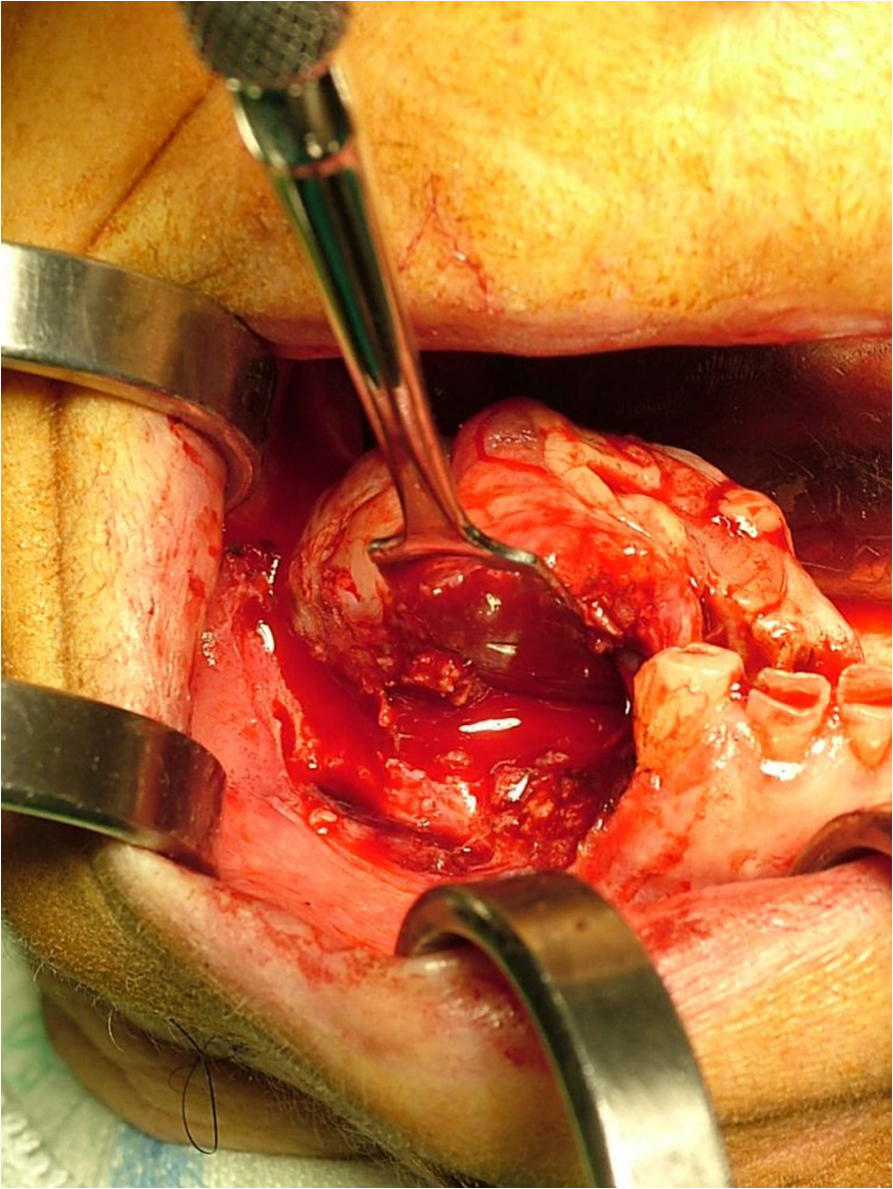
Enucleated lesion, Patient 1

**Fig. 5:**
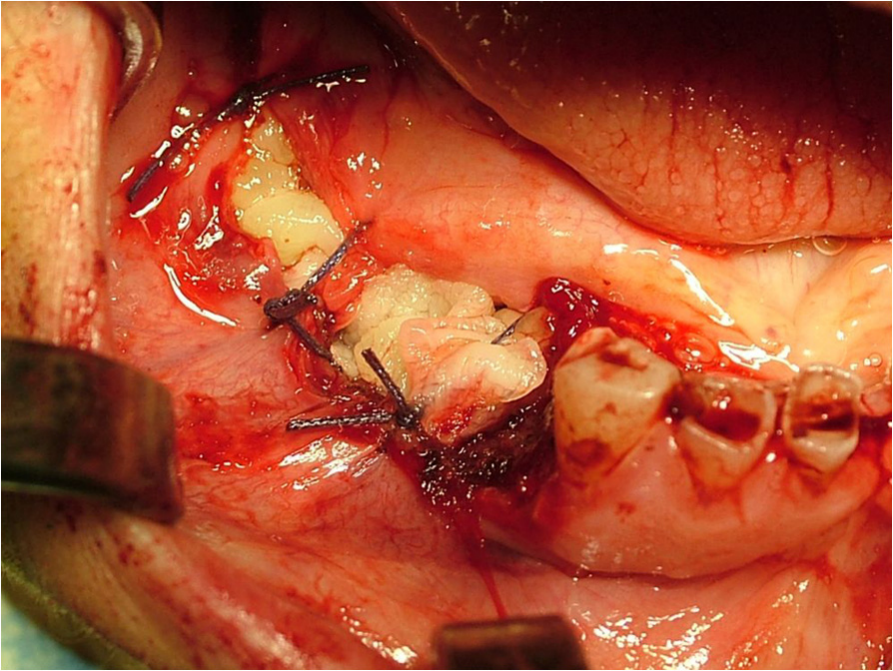
Suture and stabilization of leukocyte-platelet rich fibrin membranes, Patient 1

**Fig. 6:**
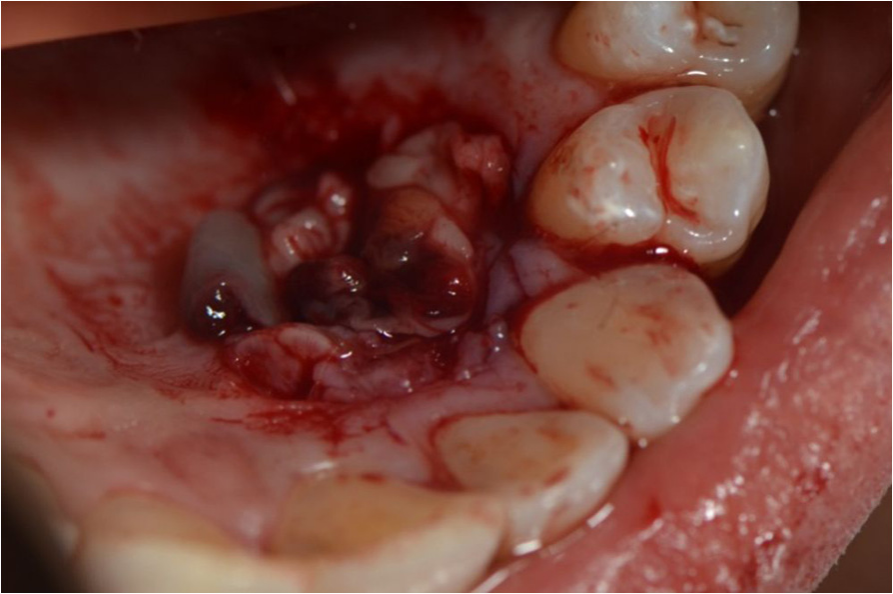
Leukocyte-platelet rich fibrin membranes on the surgical wound, Patient 2

**Fig. 7:**
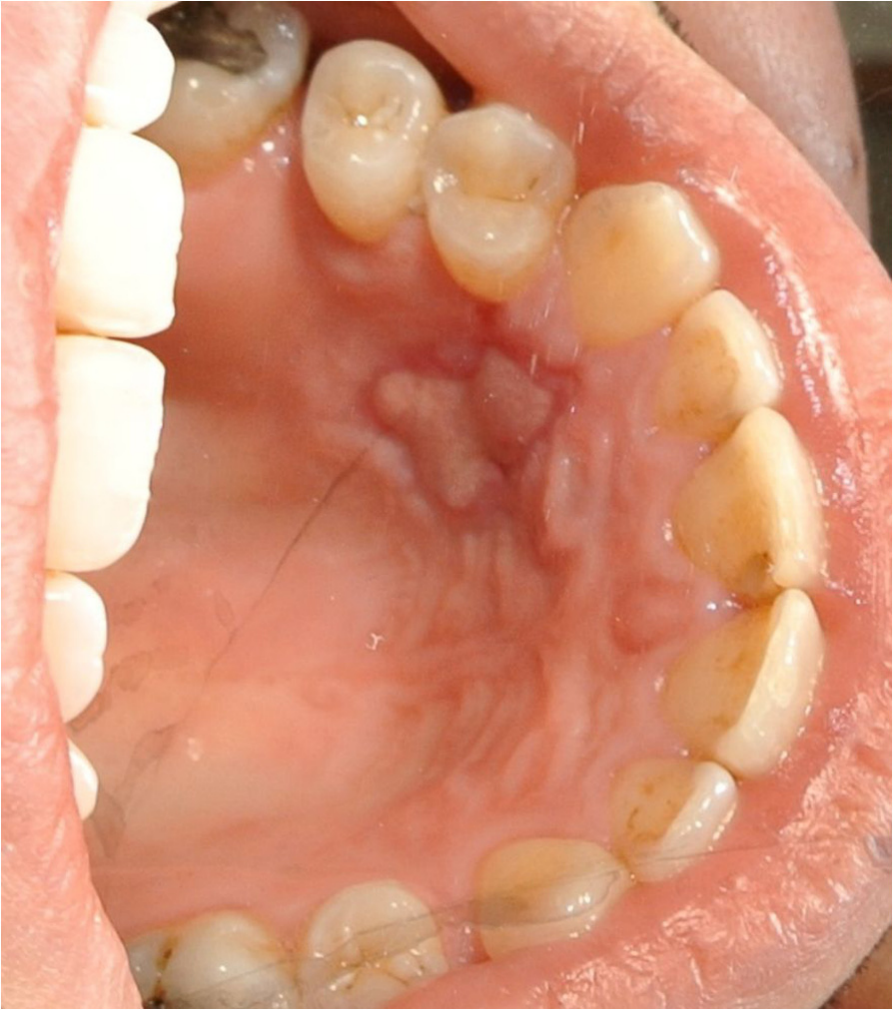
Healing, photo after 14 postoperative days, Patient 1

**Fig. 8:**
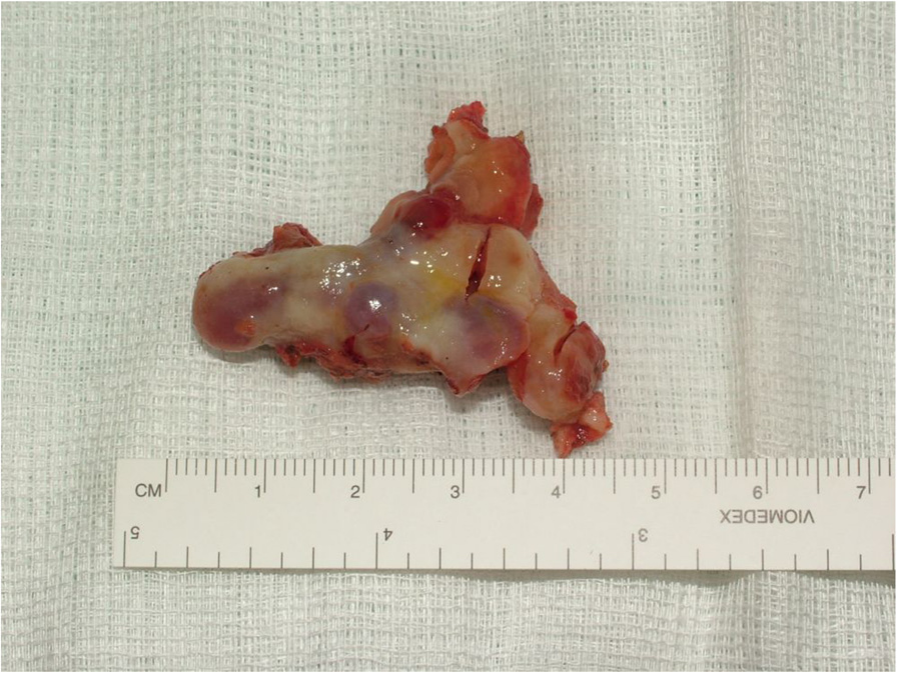
Biopsy specimen, Patient 1

**Fig. 9:**
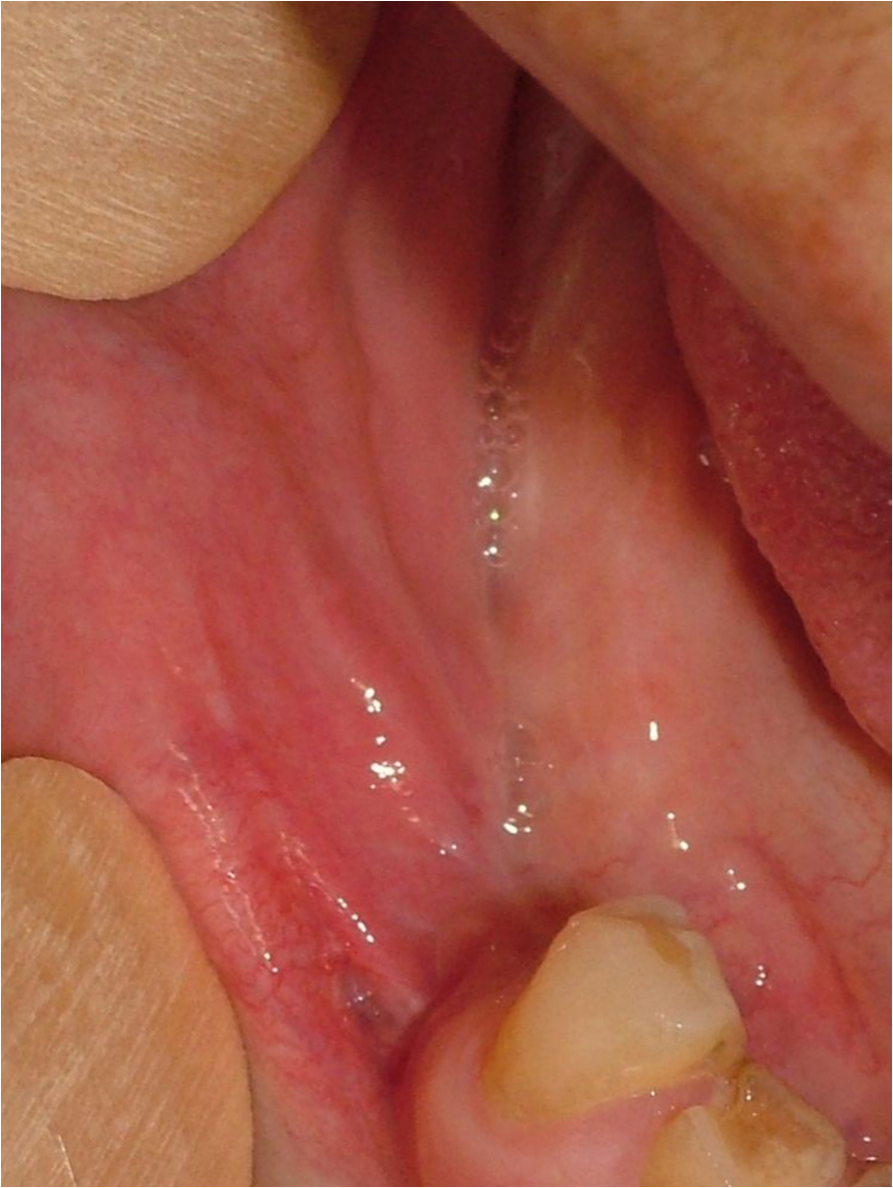
Healing, photo at 1 month, Patient 1

**Fig. 10:**
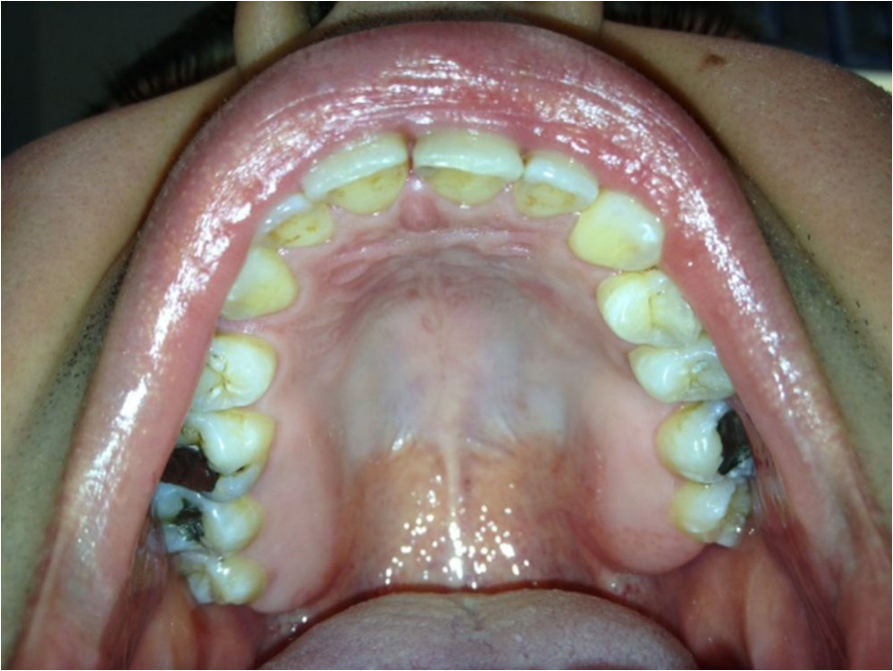
Healing, photo at 1 month, Patient 2

Prior antibiotic therapy with amoxicillin clavulanate (2g 1 hour before operation) infiltrative anesthesia (mepivacaine 30mg/mL and epinephrine 0.01mg/mL) was performed. Full thickness incisions were realized by Bard-Parker blades number 15 and they included at least 2 mm of healthy tissue. The lesions strongly adhered to the floor below. They were lifted by Prichard’s periosteal spatulas and surgical tweezers (Fig. 4). At the same time blood samples (40cc per patient) were collected in test tubes without anticoagulant to obtain the L-PRF. L-PRF protocol [9] is realized with the Intra-Spin L-PRF which provides blood centrifugation at 2700 revolutions per minute (rpm) for 12 minutes. Centrifugation determines the formation of the clot and the activation of the growth factors. After centrifugation L-PRF clots were collected together with some red blood cells from the test tubes using sterile scissors. L-PRF clots were compressed between two sterile gauzes to obtain manageable membranes useful to cover the wounds. The L-PRF membranes were stabilized with horizontal mattress and single sutures in Vicryl 3/0 (Figs. 5 and 6) removed after 7 days. After the surgery, the patients were encouraged to take, in case of pain, acetaminophen (1g/8 hours) or ibuprofen (600mg/8 hours) and a collutory with chlorhexidine 0.20% was prescribed for 7 days. Tissue healing was clinically evaluated after 1, 3, 7, 14 and 30 postoperative days (Fig. 7). Healing was quick and without dehiscences. Therefore, complete excision of the lesion was performed and the entire specimen submitted for histopathological examination (Fig. 8). Histological reports highlighted giant-cell epulis for the first case and fibro-osseous epulis for the second case. No recurrences were observed after 2 years of semi-annual follow up (Figs. 9 and 10).

## Discussion

Although the epulis in its form of giant-cell epulis and fibro-osseous epulis is a benign disease, it is important to find time to make the correct therapy. Both young and old patients must take prompt actions to avoid growth of oral lesions that can begin to become difficult to manage. There is unanimous consensus on the surgical excision of these lesions in toto to prevent recurrence. The target of these case reports is to focus on the management of the L-PRF and its placement on the surgical wounds. The first benefit is given by covering the surgical site, which would otherwise heal by secondary intention, which provides greater comfort to the patient in the postoperative period. The same wound healing appears to be substantially accelerated because of the structural and neoangiogenetical properties of L-PRF [14]. In the polymerization step of the L-PRF, thrombin, which is present in physiological concentrations, allows the formation of a fibrin matrix in a slow and natural way obtaining a flexible structure capable of determining entrapment of cytokines, cell migration and tissue healing [15]. The effect of L-PRF on reducing pain and postoperative swelling has the same importance. There are countless practices that the L-PRF offers in the dental field such as the accelerated healing of extraction sockets and use with other biomaterials in bone regeneration (GBR) [16]. In the context of tissue regeneration we evaluated the role of L-PRF in the promotion of wound healing. We also stress the importance of such a gel in the control of hemostasis in patients taking anti-platelets and/or anticoagulants for whom extractions can be performed without discontinuation of these drugs.

## Conclusions

According to the encouraging results obtained in these two clinical cases about tissue healing after application of L-PRF membranes, we suggest that L-PRF be used to cover wounds after exeresis of oral neoformations such as hyperplastic gingival lesions.

## Consent

Written informed consent was obtained from the patients for publication of this case report and any accompanying images. A copy of the written consent is available for review by the Editor-in-Chief of this journal.
